# Cloning and Functional Verification of *CmRAX2* Gene Associated with Chrysanthemum Lateral Branches Development

**DOI:** 10.3390/genes13050779

**Published:** 2022-04-27

**Authors:** Jingjing Song, Yijun Chen, Xin Li, Qiqi Ma, Qinglin Liu, Yuanzhi Pan, Beibei Jiang

**Affiliations:** College of Landscape Architecture, Sichuan Agricultural University, Chengdu 611130, China; s15982219113@163.com (J.S.); chenenyj@163.com (Y.C.); l15680825713@163.com (X.L.); maqiqi0410@163.com (Q.M.); qinglinliu@126.com (Q.L.); scpyzls@163.com (Y.P.)

**Keywords:** chrysanthemum, *CmRAX2*, lateral branches development, gene cloning

## Abstract

Chrysanthemum (*Chrysanthemum morifolium*), as one of the four major cut flowers in the world, occupies a large position in the world’s fresh cut flower market. The *RAX2* gene is an R2R3 MYB transcription factor that is associated with the development of the axillary bud. In this study, the *CmRAX2* gene cloned by homologous cloning in Chrysanthemum morifolium ‘Jinba’ is localized in the nucleus and cytoplasm, having a complete open reading frame (ORF) of 1050 bp and encoding 350 amino acids. The transactivation assay in yeast indicates that *CmRAX2* is a transcriptional activator. Quantitative Real-Time PCR (qRT-PCR) Analysis indicated that *CmRAX2* was preferentially expressed in the lateral branches and roots of *Chrysanthemum morifolium* ‘Jinba’, 14.11 and 10.69 times more than in leaves. After the overexpression vector of *CmRAX2* was constructed and transformed into *Chrysanthemum morifolium* ‘Jinba’, it was found that the number of lateral branches and plant height increased, and the emergence time of lateral branches and rooting time advanced after the overexpression of *CmRAX2*. The results showed that *CmRAX2* can promote the lateral bud development of the chrysanthemum, which provides an important theoretical basis for the subsequent molecular breeding and standardized production of the chrysanthemum.

## 1. Introduction

Chrysanthemum (*Chrysanthemum morifolium*) is one of the four largest cut flowers in the world, which occupies an important position in the world flower market [1]. With a wide variety of species, diverse strains and rich colors, chrysanthemums are widely used in cut flowers, potted plants, garden greening and landscaping. However, most potted and ground cover varieties have the disadvantage of weak natural branching, and artificial removal of the apical buds is usually needed to improve their branching in actual production, which leads to a significant increase in production costs and restricts its large-scale production [2]. Therefore, the study on the mechanism of lateral branch occurrence and regulation of chrysanthemum will provide an important theoretical basis for the molecular breeding and standardized production of chrysanthemum plants.

Using molecular biology methods to study the genes involved in regulating lateral branch development and their functions in chrysanthemum can provide a theoretical basis for chrysanthemum genetic engineering breeding. At the same time, genetic transformation can be used to breed new chrysanthemum strains with excellent characteristics. Molecular biology studies on lateral branch development mainly revolve around three important transcription factors: The GRAS family, TCP family and MYB family [3]. Among them, the MYB family, as one of the largest transcription factor families in plants [4], participates in many biological processes [5]. The MYB protein has a conservative DNA binding region at the N-terminal, the MYB structural domain, which usually contains four incomplete amino acid sequences (R groups). Each R group is a folding protein formed by approximately 50 amino acids, containing a series of highly conservative amino acid residues and interval sequences. Among them, the amino acid residue participates in the binding process with DNA in the form of helix-turn-helix (HTH). The interval sequence consists of an amino acid residue and one tryptophan residue every 18 amino acid intervals, which plays the role of a hydrophobic core, which is of great significance to maintain the structural stability of HTH [6,7]. MYB transcription factors can be divided into four categories according to the number of R groups: (1) The 1R-MYB family containing one MYB domain; (2) the R2R3 MYB family containing two MYB domains; (3) The R1R2R3 MYB family with three MYB domains; and (4) the 4R-MYB family with four MYB domains [8,9]. *MYB30* and *MYBL2* regulate plant development by interacting with *BES1*/*BZR1* to induce or reduce the expression of downstream genes and integrate BR and other signaling pathways [10]. Among plants, the R2R3 MYB family has the largest number and a wider scope of research [9,10,11,12,13,14,15]. It is highly conservative at the N end of the protein and the C end for the transcriptional activation domain, which is responsible for determining the interaction between proteins [16], participating in the process of cell development and morphogenesis, regulating metabolism, and plant stress responses. Here, we mainly focus on development-related genes.

In flower development, *AtMYB21*, *AtMYB24* [17], *AtMYB33*, *AtMYB65* [18], *AtMYB35* [19], *TDF1* and *AtMYB103*/*MS188* [20] in Arabidopsis and *CSA* in rice [21] regulate the growth of anther. *Brassica campestris BcMF28* and *AtMYB99* [22] regulate pollen development; *MYB57* [23], cucumber *GAMYB4* [24] and pepper *CaMYB108* [25] participate in the regulation of stamen development. Peach *MYB10.1* and tobacco *NtMYB305* [26] regulate flower development. In lateral branch development and formation, *AtMYB117*/*LOF1* and *AtMYB105*/*LOF2* play an important role in the development of the axillary meristem. The mutants of *LOF1* show a lack of lateral branches, while *LOF2* mutations enhance the phenotype of *LOF1* [27]. The *MYB181* gene of soybean has been overexpressed in Arabidopsis, resulting in a lateral branch increase and a height decrease [28]. *AtMYB37*/*RAX1*, *AtMYB38*/*RAX2* and *AtMYB84*/*RAX3* participate in the regulation of lateral branches meristem formation in a partially redundant way [29,30]. In the early stage of nutritional development, the formation of lateral branches is highly dependent on the function of *RAX1*. As plants mature, *RAX2* and *RAX3* mainly control the formation of lateral branches in the later stage of nutritional development. The *RAX* (Regulators of Axillary Meristems) gene is homologous with the tomato *BLIND* gene formed by earlier controlled lateral branches [31]. Under environmental stress, the expression of *AtMYB2* protein increases and binds to the promoter of the *RAX1* gene to inhibit the expression of *RAX1*, resulting in fewer branches [32]. *NtRAX2* is overexpressed in tobacco, and transgenic plants have lateral branches earlier than wild-type plants, which can promote the development of lateral branches [3]. *PhRAX2* responds to the regulation of cytokinin in *Petunia hybrida* and may indirectly participate in plant branch development as a downstream regulatory factor [33]. In the chrysanthemum, no research has been reported on the effect of the *RAX* gene on the development and regulation of lateral branches. The purpose of this study was to obtain the *CmRAX2* gene by homologous cloning, to clarify its subcellular localization, protein physical and chemical properties, transcription activation, etc., and to construct an overexpression vector to transform into chrysanthemum by the method of agrobacterium infection leaf disk, then obtaining the overexpression of *CmRAX2 Chrysanthemum morifolium* ‘Jinba’ to study the function of *CmRAX2* and its regulation on the development of chrysanthemum lateral branches.

## 2. Materials and Methods

### 2.1. Plant Materials and Cultivation Conditions

The material was taken from the *Chrysanthemum morifolium* ‘Jinba’ preserved by the School of Landscape Architecture of Sichuan Agricultural University, and the seedlings were grown in a medium supplemented with 30 g/L sucrose, 7.0 g/L agar and 4.4 g/L MS (Murashige and Skoog 1962), which was placed in a culture room with a temperature of 22–25 °C, a photoperiod of 16 h light/8 h dark, relative humidity of 65% and a light intensity of 100–120 μmol/(m^2^·s). The experiment started in April 2021.

### 2.2. Gene Cloning and Sequence Analysis

The total RNA was extracted from the leaves of a ‘Jinba’ plant using the trizol method (Vazyme Biotech Co., Ltd. Nanjing, China) and was converted into cDNA using the HiScript^®^ III 1st Strand cDNA Synthesis Kit (+gDNA wiper) reverse transcriptase (Vazyme Biotech Co., Ltd.), while the reversed cDNA was subjected to the PCR procedure (Supplementary Tables S1 and S2) by Thermal Cycler (T100, BIO-RAD), using homologous primers (Table 1) and 1×T3 Super PCR Mix, followed by sequencing.

Protparam (http://web.expasy.org/protparam/, accessed on 5 January 2022) was used to analyze the physical and chemical properties of proteins, and the conservative domain of proteins was analyzed by the CD-Search tool of the National Center for Biotechnology Information site (NCBI, https://www.ncbi.nlm.nih.gov, accessed on 5 January 2022). After that, sequences highly similar to *CmRAX2* protein sequences were retrieved in the NCBI database (https://blast.ncbi.nlm.nih.gov/Blast.cgi, accessed on 6 January 2022), and a phylogenetic tree was generated with the neighbor-joining method [34] using Molecular Evolutionary Genetics Analysis, version 10 (MEGA X) software [35,36] and constructed into a phylogenetic tree. In the Bootstrap test, the percentage of replication trees (1000 replicates) of the associated taxa clustered together is displayed next to the branch.

### 2.3. Subcellular Localization of CmRAX2

After ligating the target fragment with pBWA(V)HS-GFP by T4-ligase, the competent *Escherichia coli* (*E. coli*) was transformed and coated with Kana resistance plate. The successful vector was named pBWA(V)HS-*CmRAX2*-GLosgfp after PCR detection, sequencing verification and enzyme digestion verification. The expression vector pBWA(V)HS-*CMRAX2*- GLosgfp and the vector pBWA(V)Hs-GFP (control) containing only GFP were transformed into *Agrobacterium tumefaciens* GV3101 by the electric transfer method, coated with LB, cultured for 2 days at 30 °C, then cultured in liquid YEB for 170 r/min at 28 °C for 1 h. After centrifugation, the supernatant was discarded and re-suspended with MgCl_2_ (OD600 to 0.6). Tobacco was injected with a 1 mL syringe without the gun tip. After 2d culture under low light, the tobacco was photographed and observed under a laser focusing microscope (Zeiss, LSM510 Meta, Carl Zeiss AG). During colocalization, the marker (NLS-MKATE) was transformed into Agrobacterium, suspended together with the constructed vector plasmid Agrobacterium, mixed at a ratio of 1:1 before injection and then injected into tobacco leaves. After culturing in low light for 2 d, the leaves were photographed and observed under a laser focusing microscope [37]. The chloroplast fluorescence signal excitation wavelength was 640 nm, the emission wavelength was 675 nm, the green fluorescent protein GFP excitation wavelength was 488 nm and the emission wavelength was 510 nm.

### 2.4. Analysis of the Transcriptional Activation Activity of CmRAX2

Yeast Y2H Gold cells were coated on a YPDA solid medium and placed upside down in a 30° incubator until single colonies grew. Single colonies were picked using a 10 µL white tip and punched together into 5 mL of the YPDA liquid medium and incubated at 250 rpm for 16 h at 30 °C. Next, 300–500 µL of the bacterial solution was aspirated into 50 mL of the liquid YDPA medium and incubated at 30 °C for 8–12 h at 250 rpm until the OD600 value was 0.4–0.6. We then centrifuged at 2000 rpm for 5 min and discarded the supernatant, taking 30 mL of the pre-cooled deionized water to resuspend the bacteria. It was then centrifuged at 2000 rpm for 5 min and the supernatant was discarded, taking 1.5 mL of pre-cooled TE/LiAc to resuspend the bacteria. It was then centrifuged at 12000 rpm for 15 min and the supernatant was discarded, taking 1.5 mL of pre-cooled TE/LiAc to resuspend the bacteria. The supernatant was discarded after centrifugation at 2000 rpm for 5 min, and 1.5 mL of pre-chilled TE/LiAc was used to resuspend the bacteria, and then the bacteria were placed on ice.

Y2H GOLD yeast competent cells were used for the Self-activation assay of bait recombinant plasmids. The carrier DNA was boiled in boiling water at 100 °C for 5 min and immediately put on ice for 2 min, and this was repeated once. Taking 1.5 mL of a sterile EP tube, we added 50 μL yeast receptor cells + 5 μL carrier DNA + 100 ηg decoy plasmid + 100 ηg prey empty to prepare the transformation system. We then added 500 μL of PEG/LiAc (8 mL 50% PEG + 1 mL LiAc + 1 mL TE), mixed it well and incubated it at 30 °C for 30 min, mixing every 10 min. Then, 20 μL of DMSO was added and mixed gently, incubating it at 42 °C for 15 min and mixing it every 5 min. After being centrifuged for 1 min, we removed the supernatant, added 800μL of YPDA resuspension and sealed it with sealing film, followed by incubation at 30 °C and 150 rpm for 1.5 h. Then, we centrifuged it for 5 min, discarded the supernatant, added 1 mL of 0.9% NaCl to resuspend it and took 150μL to coat the plate. This was then incubated at 30 °C for 4–5 days in an inverted position, and we observed the colony diameter and color.

### 2.5. Quantitative Real-Time PCR Analysis

The total RNA of different tissues was extracted by the trizol method (Vazyme Biotech Co., Ltd. Nanjing, China), and the RNA concentration, as well as OD260/OD280 and OD260/OD230, were measured using a microplate reader, while the integrity of RNA was detected by agarose gel electrophoresis. The total RNA was converted into cDNA using the TransScript^®^ II All-in-One First-Strand cDNA Synthesis SuperMix for qPCR (One-Step gDNA Removal) (TransGen Biotech, Beijing), according to the manufacturer’s protocol. Using Actin with a length of 699 bp (AB205087) [38] as an internal parameter, Quantitative Real-Time PCR (qRT–PCR) (CFX Connect, BIO-RAD, Hercules, CA, USA) was introduced to analyze the expression levels of *CmRAX2* in different tissues of ‘Jinba’. Specific primers were designed using Primer 5.0 (Table 1). The formula 2^−∆∆Ct^ was used to calculate their values. The conditions of reverse transcription PCR (RT-PCR) and qRT-PCR can be found in the supplementary table (Supplementary Tables S3–S6). The results were described using standard errors by SPSS software. 

### 2.6. Construction of Expression Vector and Overexpressing CmRAX2 in the ‘Jinba’

The SmaI enzyme cuts pBI121 carriers, recombines the amplified ORF fragment with linearized carriers and the 35S promoter was inserted to drive it (Supplementary Figure S1), transforming *Escherichia coli*. The overexpression transgenes were inserted into *Agrobacterium tumefaciens* GV3101 using the freezing transformation method. Then ‘Jinba’ were transformed using the agrobacterium infection of the chrysanthemum leaf disk. Leaf discs were transferred to the selection medium with kanamycin 10 mg/L, and the first selection culture was carried out for 14 d. After the healing tissues grew resistant buds, the resistant buds were cut and inoculated into the rooting medium and screened again using 7 mg/L kanamycin, while the rooted seedlings were resistant plants. DNA was extracted by the method of CTAB, then subjected to the PCR procedure (Supplementary Tables S1 and S2). The primers *35S*-F and *CmRAX2*-R were used in the PCR procedure, which could obtain a product length of approximately 1150. The RNA of resistant plants was extracted and reverse transcribed into DNA, and the expression levels of *CmRAX2* in different plants were analyzed by qRT-PCR. The test method was the same as above. Three overexpression lines and wild types were selected to count the average plant height and average numbers of lateral branches, with 3 for each line and 3 replicates. The results were described using standard errors. Statistics and analyses of data were performed using SPSS software.

## 3. Results

### 3.1. Isolation and Sequence Analysis of CmRAX2 from ‘Jinba’

**Figure 1:**
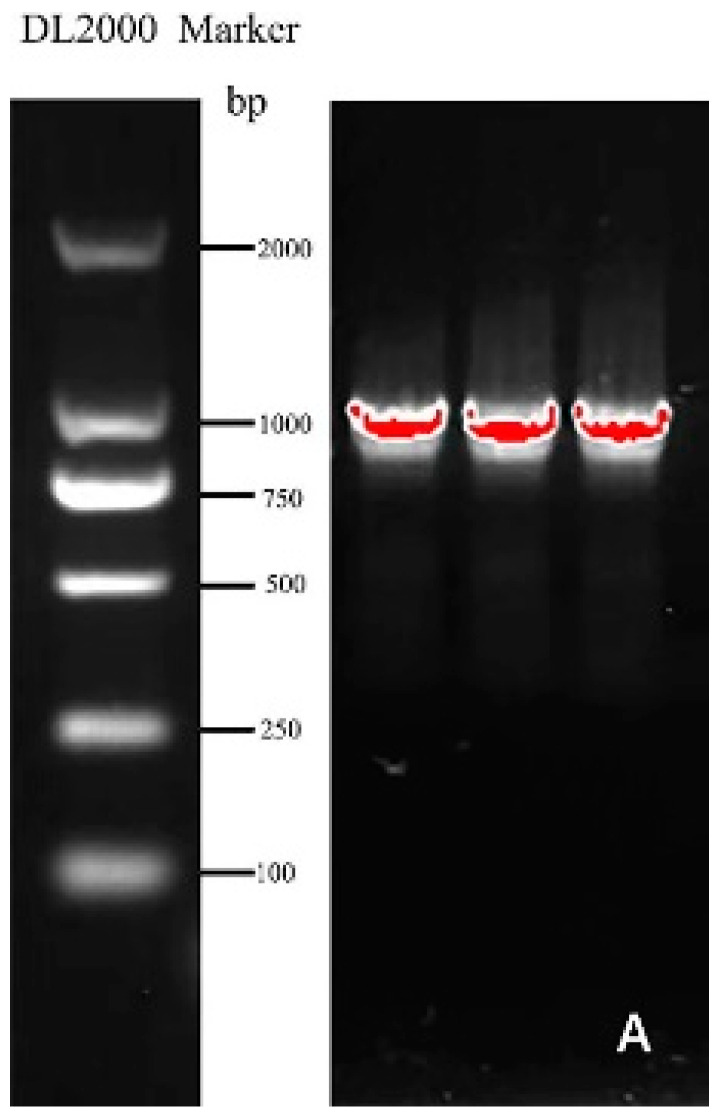

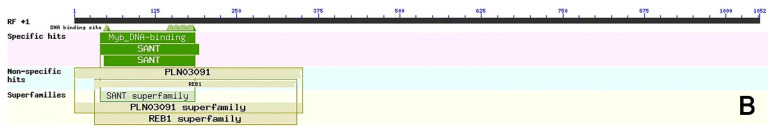
(**A**) Gene cloning figure. (**B**) Conservative domain analysis.

Specific primers (Table 1) were designed according to the mRNA sequence of the *Helianthus annuus* transcription factor *RAX2* gene (ID: XM_022123835.2) from NCBI and employed to clone the gene named *CmRAX2* (Figure 1A). The total ORF length of the *CmRAX2* gene is 1050 bp, and 350 amino acids are encoded. Through online analysis of ProtParam (https://web.expasy.org/protparam/, accessed on 5 January 2022), the isoelectric point of *CmRAX2* is 8.01 and the molecular weight is 39505.74. The instability index is computed to be 58.42, which classifies the protein as unstable. The grand average of hydropathicity is −0.621, which makes it a hydrophilic protein. The CD-Search analysis shows that *CmRAX2* has an MYB binding domain, and through comparison, it is found that *CmRAX2* belongs to the R2R3 family (Figure 1B).

### 3.2. Sequences Alignment and Phylogenetic Analysis of CmRAX2

**Figure 2:**
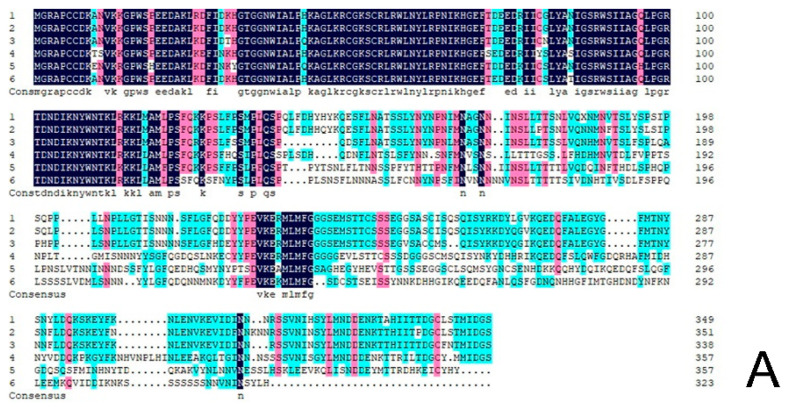

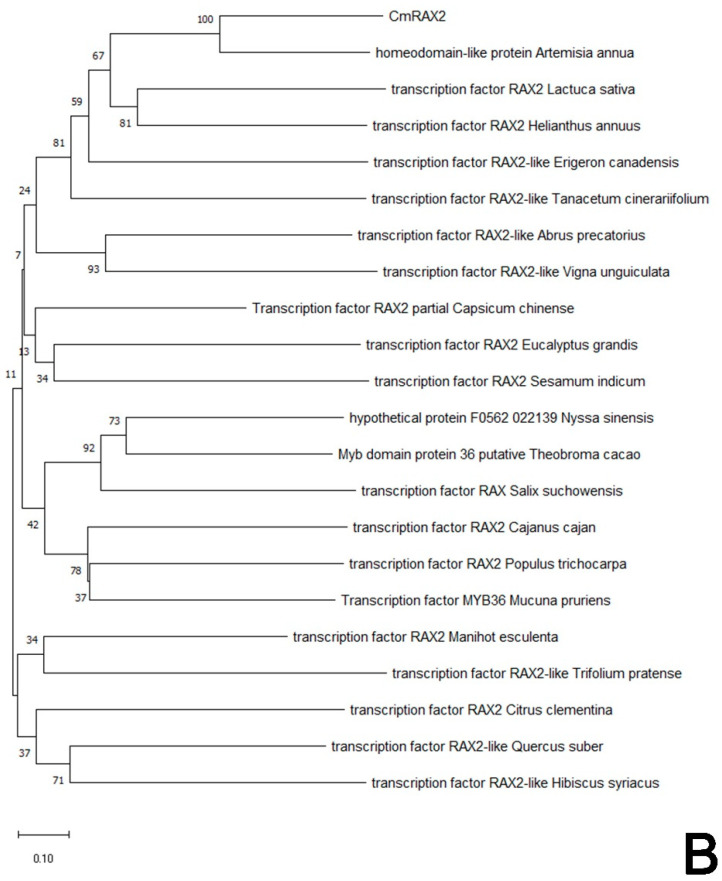
(**A**) Sequence alignment. (**B**) Phylogenetic tree.

The protein sequence of *CmRAX2* was submitted to NCBI for blast. Homologous sequences similar to the *CmRAX2* protein were downloaded, and the homology of the *CmRAX2* protein with the *RAX2* protein sequence in other species was compared using DNA MAN software (Figure 2A). The results showed that the *CmRAX2* protein in chrysanthemum ‘Jinba’ was 94.03% similar to the R2R3 MYB protein in *Artemisia annua*, 82.62% similar to the *pyrethrum cinerariifolium RAX2* protein, and 60.86%, 58.93% and 56.04% similar to *Lactuca sativa*, *Helianthus annuus* and *Erigeron canadensis RAX2*. The *CmRAX2* protein and *RAX2* proteins in other species contain two MYB domains, which are R2R3-like MYB transcription factors. The amino acid sequence of *CmRAX2* is highly conserved with other *RAX2* proteins in the MYB-binding domain, and the first tryptophan in the R3 MYB domain is replaced by alanine.

In addition, to determine the evolutionary relationship between *CmRAX2* in chrysanthemum and other plant species, the phylogenetic tree was constructed with the neighbor-joining method by MEGA X following multiple alignments of protein sequences (Figure 2B). The alignment results showed that the *CmRAX2* protein sequence has consistency in different plants and the *CmRAX2* protein has the highest evolutionary similarity with *Artemisia annua* (GEY50283.1).

### 3.3. Subcellular Localization of CmRAX2

**Figure 3:**
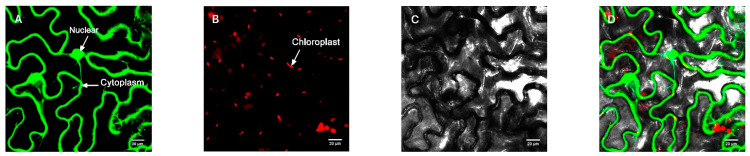
Empty vector control. (**A**) Fluorescence channel. (**B**) Chloroplast fluorescence channel. (**C**) Bright field. (**D**) Superimposed plot.

**Figure 4:**
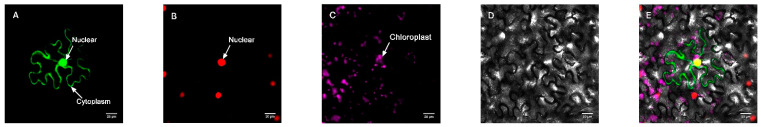
Target protein. (**A**) Fluorescence channel. (**B**) Marker fluorescence channel. (**C**) Chloroplast fluorescence channel. (**D**) Bright field. (**E**) Superimposed plot.

In order to further determine the subcellular localization of the protein, the pBWA (V) HS-GLosgfp expression vector was constructed by fusing the ORF sequence of *CmRAX2* with the N-terminal of GFP fluorescent protein to transform tobacco leaves, and an empty vector was used as the control. As shown in Figure 3 and Figure 4, the *CmRAX2* gene in tobacco leaves was not only consistent with the expression location of the nuclear marker, but was also located in the cytoplasm with good transient expression intensity.

### 3.4. Analysis of the Transcriptional Activity of CmRAX2

**Figure 5:**
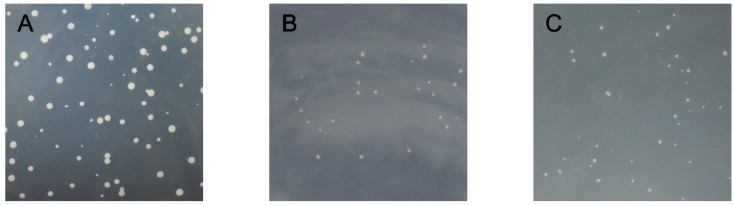
(**A**) DDO plate. (**B**) TDO plate. (**C**) QDO plate.

As shown in Figure 5, the bait plasmid PGBKT7-*CmRAX2* and prey unloaded PGADT7 co-transformed the Y2H Gold Competent Cell, which was able to grow after being coated on a DDO plate. This indicated that the recombinant bait plasmid was successfully transferred into host bacteria and had no toxicity to host bacteria. The bait protein could grow on the TDO plate, indicating that the bait protein could activate the expression of reporter gene *His3* in yeast cells. The QDO plate was coated, and the decoy protein was found to activate the expression of *ADE2*. Point-to-point verification of the two-hybrid yeast showed that the recombinant bait plasmid could activate the expression of Y2H Gold reporter genes *His3* and *ADE2*, indicating that *CmRAX2* is a transcriptional activator.

### 3.5. Expression Level Analysis of CmRAX2 in ‘Jinba’

**Figure 6:**
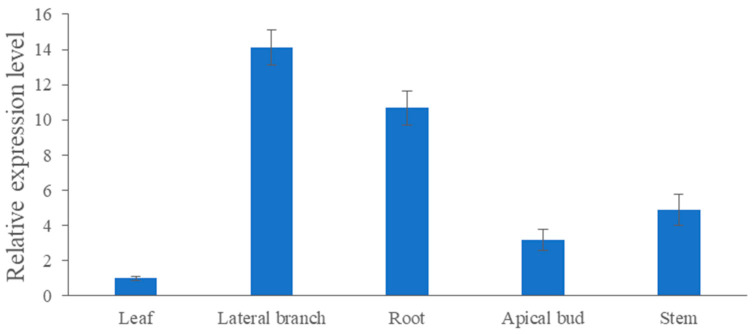
Expression-level analysis of *CmRAX2* in ‘Jinba’. The lines are used to show standard errors.

As shown in Figure 6, the expression level of *CmRAX2* varies greatly in different organs. The expression in lateral branches and roots is 14.11 and 10.69 times higher than the expression in leaves, respectively, while the expression in stems and apical buds is 4.88 and 3.17 times lower than that in leaves. *CmRAX2* exists in various organs of plants, but it has significant tissue specificity.

### 3.6. Genetic Transformation of CmRAX2 Gene in ‘Jinba’

**Figure 7:**
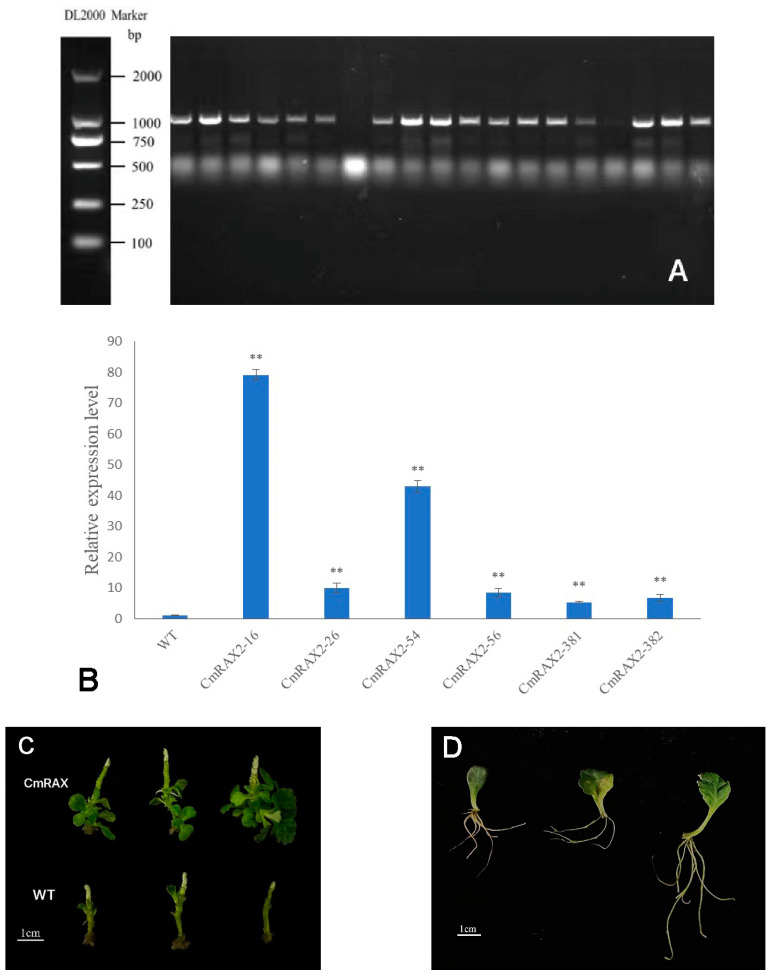
(**A**) PCR detection of kanamycin-resistant plants. (**B**) qRT-PCR of kanamycin-resistant plants. The lines are used to show standard errors. The ** means significantly higher than wild-type plants. (**C**) Transgenic chrysanthemum and wild-type plants 30d after transplantation. (**D**) Leaf rooting.

**Figure 8:**
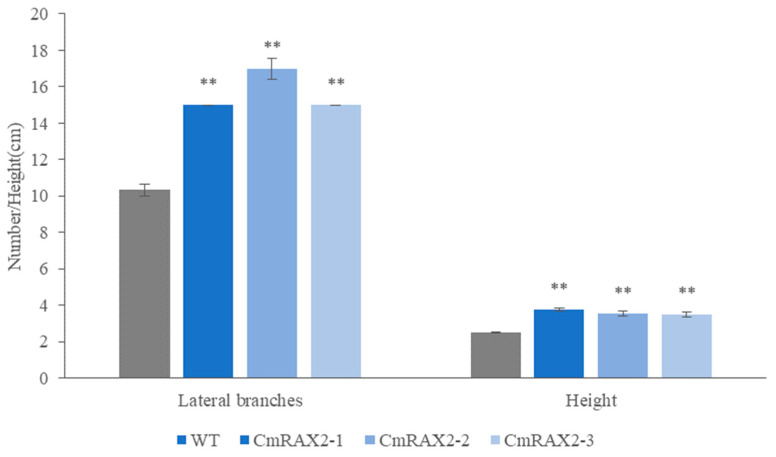
Effect of *CmRAX2* on the number of lateral branches and plant height of chrysanthemum ‘Jinba’. The lines are used to show standard errors. The ** means significantly higher than wild-type plants.

The overexpression vector was transformed into chrysanthemum by *Agrobacterium tumefaciens*, and transgenic plants were obtained after culture and screening (Figure 7A–C). Since the *CmRAX2* gene is an endogenous gene of chrysanthemum ‘Jinba’, the 35S promoter carried in the vector was used as a marker, and 35S-F and *CmRAX2*-R were used as specific primers for screening. The expression level of *CmRAX2* was determined for the plants obtained by screening (Figure 7B). The expression level of *CmRAX2*-16 was the highest, which was 79 times that of WT, followed by *CmRAX2*-54, which was 43 times that of WT. The same batch of transgenic and wild plants with the same growth status had the apical buds removed at the same time. After the lateral branches had grown, they were transplanted into the MS medium for phenotype observation (three biological treatments were repeated as one treatment, with three treatments in total). The observation showed that the transgenic plants started rooting 4 days after transplanting, 5 days earlier than the wild-type plants, and leaves also rooted naturally when dropped into the MS medium (Figure 7C,D). In addition, axillary buds appeared 5 days after transplanting them into transgenic plants, while the wild-type plants were observed for the first time on the ninth day. After 30 days of growth of the chrysanthemum seedlings, we counted the number of lateral branches and measured the plant height of transgenic chrysanthemum and wild-type plants (Figure 7C). It was found that the average height of transgenic chrysanthemum was 1 cm higher than that of wild-type plants, and the average number of lateral branches was 15.67, while that of wild-type plants was only 11.00. There is a significant difference between the height and lateral branch number (Figure 8). These results indicated that the overexpression of *CmRAX2* enhanced the meristem capacity of the chrysanthemum ‘Jinba’ meristem and promoted the formation of lateral branches during plant development.

## 4. Discussion

### 4.1. CmRAX2 Is Expressed in the Nucleus and Cytoplasm as a Transcriptional Activator

The subcellular localization results showed that *CmRAX2* is expressed in the nucleus and cytoplasm. The localization of *CmRAX2* protein in the nucleus is consistent with its function as a transcription factor regulating gene transcription in the nucleus, but the results showed that *CmRAX2* protein is also expressed in the cytoplasm. A related study found that *EIN2* protein in plants could not be phosphorylated by its downstream proteins after binding to ethylene receptors on the endoplasmic reticulum membrane. In this case, a break in the intermediate position of the *EIN2* protein occurs, and the C-terminal end of the break with a nuclear localization signal is able to enter the nucleus, acting as a transcription factor in combination with *EIN3* [39]. The plant ultraviolet receptor *UVR8* is localized in the nucleus and cytoplasm, and it was suggested that it is transferred to the nucleus by the transcription factor *COP1* with a nuclear export signal [40,41,42]. The transcription factor *PIF7* in Arabidopsis is able to be activated in the nucleus after receiving light signals, functions and then dephosphorylates, and is then recognized by 14-3-3 proteins to be re-transported out of the nucleus [43]. Therefore, we analyzed the protein sequence of *CmRAX2*, and the results showed that *CmRAX2* does not have a transmembrane structure, signal peptide or nuclear localization signal. It cannot penetrate the membrane by itself but may initially be expressed in the cytoplasm or be transported out of the nucleus by other protein sequences with a nuclear export signal, such as *UVR8*. The yeast two-hybrid experiment showed that *CmRAX2* has transcriptional activation activity in yeast and acts as a transcriptional activator when expressed in chrysanthemum ‘Jinba’.

### 4.2. RAX2 Expression Patterns Vary in Different Plants

The expression patterns of the *RAX2* were analyzed in both tobacco and petunia. The qRT-PCR results showed that the transcripts of the double-copy genes *NtRAX2-S* and *NtRAX2-T* were detected in tobacco roots, stems, leaves, stem tips, axillary buds, flower buds and flowers. The expression pattern of *NtRAX2-S* in tobacco showed a general trend of flower > flower bud > axillary bud > stem tip > leaf > root > stem. The expression pattern of *NtRAX2-T* was similar to *NtRAX2-S*, showing a general form of flower > axillary bud > flower bud > stem tip > leaf > root > stem. Both have the highest expression in tobacco floral organs and the lowest expression in stem segments, with only a small number of transcripts [3]. In petunia, *PhRAX2* was expressed in the stem tip, root, leaf axil, stem segment and leaf, with the highest expression in the stem tip, followed by the leaf axil, and the lowest expression in the stem segment, with an overall expression of stem tip > leaf axil > leaf > root > stem [33]. In chrysanthemum ‘Jinba’, we found that *CmRAX2* had the highest expression in lateral shoots, followed by roots, stem segments and terminal shoots, and the lowest expression in leaves. This indicates that the expression pattern of *RAX2* varies in different plants, but it is highly expressed in the meristematic parts such as flowers, stem tips, roots and lateral shoots in tobacco, petunia and cut flower chrysanthemum ‘Jinba’, indicating that *RAX2* may play an important regulatory role in the initiation and development of meristematic tissues.

### 4.3. RAX Plays a Role in Promoting the Development of Lateral Bud

In the early stage of plant vegetative development, the formation of lateral buds is highly dependent on the function of *RAX1*, and this dependence diminishes as the plant matures, while *RAX2* and *RAX3* play a more important role in the middle and late stages of plant nutritional development [29,30]. Overall, *RAX1* and *RAX2* play a greater role in plant lateral bud development than *RAX3*, but the proteins encoded by all three are functionally redundant, and knockout can reduce the number of lateral buds and meristem formation in plants [3,44].

In Arabidopsis, external stress can increase the expression of the *AtMYB2* protein and bind the promoter of *RAX1* to repress the expression of *RAX1*, resulting in the inhibition of the lateral bud development process [32]. *AtRAX1* acts as a transcriptional activator in the early stages of axillary meristem development and regulates the expression of *CUC2* in the axillary center region, thus predicting the future axillary meristem location. Meanwhile, *CUC2* is mainly involved in the regulation of lateral bud development by regulating the downstream gene *LAS* of *RAX3* in the early to middle stages of plant growth [45,46]. *AtRAX2* was shown to affect the formation of lateral buds during inflorescence development in Arabidopsis, and the overexpression of *RAX3* increased the number of lateral buds in plants [29,30]; in tobacco, *NtRAX2* was able to promote axillary bud formation in axillary meristematic tissues [3]. In petunia, the expression of *PhRAX2* indicated that *PhRAX2* can respond to the exogenous application of 6-BA CKs, but its response is not rapid. It may be that CKs do not directly promote the expression of the gene *PhRAX2* in this process, but rather act as a signal molecule to indirectly regulate the expression of downstream *PhRAX2* [33]. In this study, by comparing the phenotypes of *CmRAX2*-overexpressing transgenic plants and wild-type plants in chrysanthemum ‘Jinba’, we found that the height and number of lateral branches of transgenic plants were significantly different from those of wild-type plants. The overexpression of *CmRAX2* was able to increase the plant height, the number of lateral branches and the rooting ability of the plants. Moreover, *CmRAX2* could greatly enhance the rooting ability of the leaves in chrysanthemum ‘Jinba’. This suggested that *CmRAX2* may improve the rooting ability of chrysanthemum ‘Jinba’, facilitating the cell proliferation of meristematic tissues, the initiation of axillary meristematic tissues and the formation of axillary buds. We speculate that *RAX* may be involved in the germination of axillary tissue by participating in the downstream regulatory pathway of cytokinin.

In the MYB gene family, members of the same subfamily are able to act as upstream transcription factors to regulate the same downstream target genes, but their regulatory mechanisms are not exactly the same in different species. MYB transcription factors within the same subfamily can also regulate the same metabolic pathways in different cell types, but their gene expression patterns are not identical [47,48]. *RAX1*, *RAX2* and *RAX3* belong to the same subgroup 14 of the MYB protein family, all of which are involved in regulating plant lateral bud development, play a role in promoting the plant meristem and are functionally redundant. Therefore, we speculated that in chrysanthemum, *CmRAX2* may be involved in chrysanthemum lateral bud development by regulating the expression of *CUC2* and *LAS* as a transcription factor, just as *AtRAX1* and *RAX3* do.

## 5. Conclusions

The *CmRAX2* gene, which is located in the nucleus and cytoplasm and is associated with lateral branch development, was identified and cloned. The overexpression of *CmRAX2* in chrysanthemum ‘Jinba’, which acts as a transcriptional activator, increased the number of lateral branches and plant height. We speculate that in chrysanthemums, *CmRAX2* may regulate the development of lateral branches of chrysanthemum in the later stage of nutritional development by regulating the target genes *CUC2* and *LAS*, which provides an important theoretical basis for the subsequent molecular breeding and standardized production of chrysanthemum.

## Figures and Tables

**Table 1:** Specific primers.

Gene	Primers
*CmRAX2*-F	ATGGGAAGAGCTCCTTGTTG
*CmRAX2*-R	ATTACTCCCATCAATCATAG
*35S*-F	GACGCACAATCCCACTATCC
qRT–PCR *CmRAX2*-F	TGGTACTGGTGGTAACTGGATTGC
qRT–PCR *CmRAX2*-R	AGCCATCTCAACCTGCAACTCTTG
qRT–PCR *Actin*-F	ACAACTGCTGAACGGGAAAT
qRT–PCR *Actin* -R	AATCATAGACGGCTGGAAAAG

## Data Availability

Data are contained within the article.

## Supplementary Materials

The following supporting information can be downloaded at: www.mdpi.com/article/10.3390/genes13050779/s1, Figure S1: Vector construction map; Table S1: PCR system; Table S2: PCR reaction program; Table S3: Genome DNA removal; Table S4: Reverse transcription reaction system; Table S5: qRT-PCR system; Table S6: qRT-PCR reaction program.
