# Supplementary figures and images for: Cloning and Functional Verification of CmRAX2 Gene Associated with Chrysanthemum Lateral Branches Development
Source: Genes (Basel). 2022 Apr 27;13(5):779. doi: 10.3390/genes13050779 (PMC9140354; doi:10.3390/genes13050779)

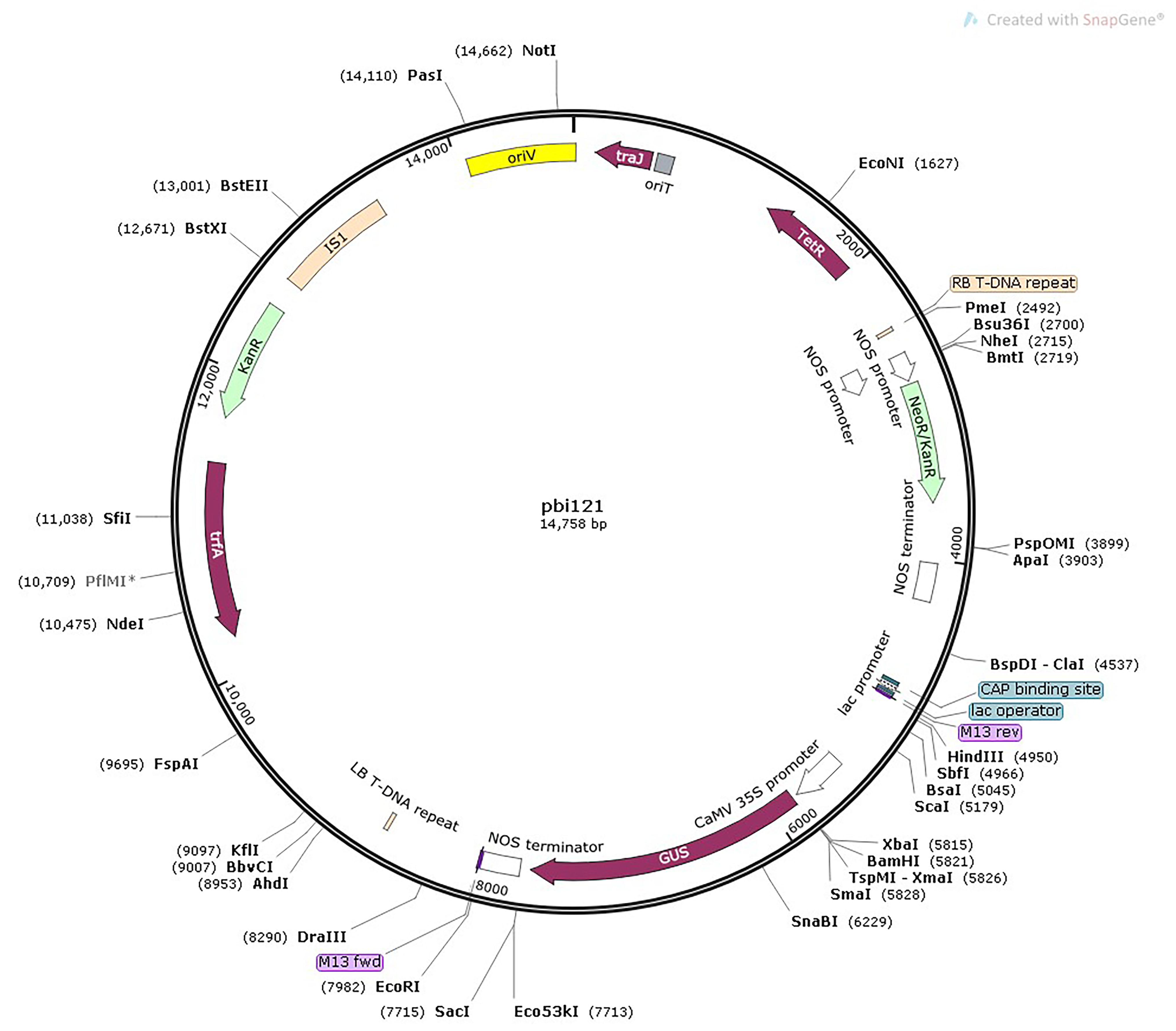

Supplement: Supplementary file 1 [file genes-13-00779-s001.zip › Supplementary Materials/supplementary figure s1.jpg]
